# Serum Levels of Substance P and Mortality in Patients with a Severe Acute Ischemic Stroke

**DOI:** 10.3390/ijms17060991

**Published:** 2016-06-22

**Authors:** Leonardo Lorente, María M. Martín, Teresa Almeida, Antonia Pérez-Cejas, Luis Ramos, Mónica Argueso, Marta Riaño-Ruiz, Jordi Solé-Violán, Mariano Hernández

**Affiliations:** 1 Intensive Care Unit, Hospital Universitario de Canarias, Ofra, s/n. La Laguna, Tenerife 38320, Spain; 2 Intensive Care Unit, Hospital Universitario Nuestra Señora Candelaria, Crta Rosario s/n, Santa Cruz Tenerife 38010, Spain; mar.martinvelasco@gmail.com; 3 Unidad de Genética, Instituto de Enfermedades Tropicales y Salud Pública de Canarias, Universidad de La Laguna, Campus de Anchieta, Avda. Astrofísico Francisco Sánchez s/n, La Laguna, Tenerife 38071, Spain; tacosalm@ull.edu.es (T.A.); mnhdez@ull.edu.es (M.H.); 4 Laboratory Department, Hospital Universitario de Canarias, Ofra, s/n. La Laguna, Tenerife 38320, Spain; aperezcejas@gmail.com; 5 Intensive Care Unit, Hospital General La Palma, Buenavista de Arriba s/n, Breña Alta, La Palma 38713, Spain; lramosgomez@gmail.com; 6 Intensive Care Unit, Hospital Clínico Universitario de Valencia, Avda. Blasco Ibáñez nº17–19, Valencia 46004, Spain; moni_begasa@hotmail.com; 7 Servicio de Bioquímica Clínica, Complejo Hospitalario Universitario Insular Materno-Infantil, Plaza Dr. Pasteur s/n, Las Palmas de Gran Canaria 35016, Spain; mriarui@gobiernodecanarias.org; 8 Intensive Care Unit, Hospital Universitario Dr. Negrín, CIBERES. Barranco de la Ballena s/n, Las Palmas de Gran Canaria 35010, Spain; jsolvio@gobiernodecanarias.org

**Keywords:** substance P, stroke, mortality, cerebral infarction

## Abstract

Substance P (SP), a member of tachykinin family, is involved in the inflammation of the central nervous system and in the appearance of cerebral edema. Higher serum levels of SP have been found in 18 patients with cerebral ischemia compared with healthy controls. The aim of our multi-center study was to analyze the possible association between serum levels of SP and mortality in ischemic stroke patients. We included patients with malignant middle cerebral artery infarction (MMCAI) and a Glasgow Coma Scale (GCS) lower than 9. Non-surviving patients at 30 days (*n* = 31) had higher serum concentrations of SP levels at diagnosis of severe MMCAI than survivors (*n* = 30) (*p* < 0.001). We found in multiple regression an association between serum concentrations of SP higher than 362 pg/mL and mortality at 30 days (Odds Ratio = 5.33; 95% confidence interval = 1.541–18.470; *p* = 0.008) after controlling for age and GCS. Thus, the major novel finding of our study was the association between serum levels of SP and mortality in patients suffering from severe acute ischemic stroke.

## 1. Introduction

Ischemic stroke is associated with considerable resource consumption, disability, and mortality [1]. In cerebral infarction, cell death occurs due to vascular obstruction and the restriction of blood that contains oxygen and substrates for neurons. In addition, there may be also secondary injury (due to different mechanisms, such as inflammation, apoptosis, oxidative stress, increased vascular permeability, and cerebral edema), which could contribute to increased cell death [2,3].

The tachykinin family includes different members as substance P (SP), neurokinin B (NKB), neurokinin A (NKA), and endokinins [4,5,6,7,8,9]. To date, three tachykinin receptors have been identified (NK1R, NK2R, and NK3R). SP and endokinins showed a preferential binding to NK1R, NKB to NK3R, and NKA to NK2R, respectively. Tachykinins are distributed throughout the peripheral and central nervous systems, and also throughout the respiratory system, gut, urinary system, blood vessels, and immune system. Tachykinins are involved in several biological processes such as inflammation, vasodilation, plasma protein extravasation, smooth muscle contraction, transmission of nociceptive responses, airway contraction, and salivary secretion. SP has been implicated in different diseases such as inflammatory bowel disease, asthma, psoriasis, anxiety, migraine, emesis, psychosis, and central and peripheral nervous systems injury [4,5,6,7,8,9].

In addition, SP could play a role in ischemic stroke: there are data suggesting that SP is involved in neurogenic inflammation, classical inflammation, and thrombosis [2,3]. Animal models of cerebral ischemia have shown a higher expression of SP in ischemic cerebral tissue [10,11,12]. However, SP has been scarcely explored in ischemic stroke patients [13,14]. In one study, higher SP-immunoreactive fiber expression was found in the brain stem of four infants with brain-stem infarction [13]. Another study with 18 patients showed higher serum SP concentrations in those with cerebral ischemia compared with healthy controls [14]. The aim of our study was to analyze the possible association between serum levels of SP and mortality at 30 days in ischemic stroke patients.

## 2. Results

**Figure 1:**
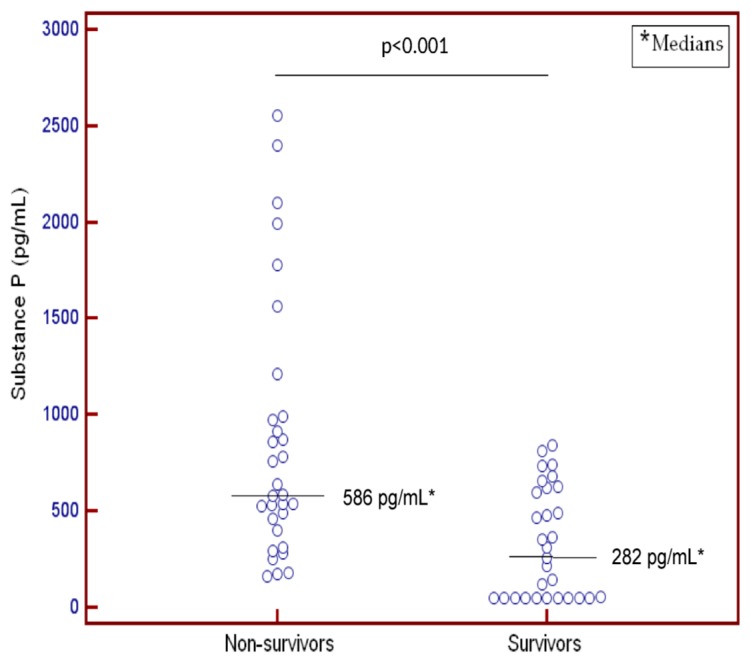
Dot-plot of serum levels of substance P in 30-day survivor and non-survivior patients.

We found that 31 of 61 severe MMCAI patients died within 30 days of diagnosis. In addition, we found that non-survivor patients had lower GCS and platelet count, higher serum levels of SP (Figure 1), and a tendency toward a higher age than surviving MMCAI patients (Table 1).

**Figure 2:**
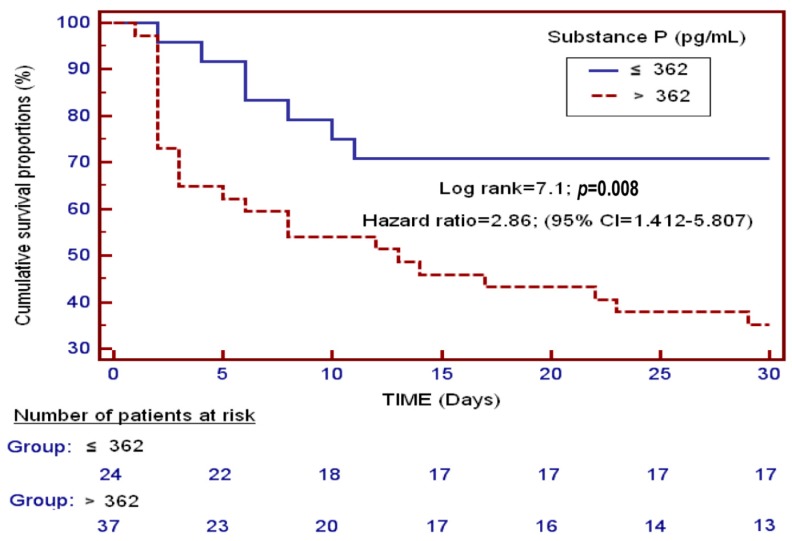
Survival curves at 30 days with serum substance P levels lower and higher than 362 pg/mL.

We found an area under the curve of 78% (95% confidence interval = 65%–87%; *p* < 0.001) for the prediction of mortality at 30 days according to serum concentrations of SP. Survival analysis showed that patients showing serum concentrations of SP higher than 362 pg/mL had a higher 30-day mortality rate than patients with lower concentrations (Hazard ratio = 2.86; 95% confidence interval = 1.412–5.807; *p* = 0.008) (Figure 2).

Regression analysis showed an association between serum levels of SP higher than 362 pg/mL and mortality at 30 days (Odds Ratio = 5.33; 95% confidence interval = 1.541–18.470; *p* = 0.008) after adjusting for GCS and age (Table 2).

We did not found a significant association between serum concentrations of substance P and TNF-alpha (rho = 0.24; *p* = 0.10).

## 3. Discussion

To the best of our knowledge, this is the largest study reporting data on serum concentrations of SP in ischemic stroke patients. The major novel findings were that non-surviving patients with severe MMCAI had higher serum concentrations of SP than survivors, and that an association between serum concentrations of SP and mortality exists in MMCAI patients.

Previously, a higher expression of SP has been found in ischemic cerebral tissue in animal models [10,11,12] and in four infants with brain-stem infarction [13]. In addition, higher serum levels of SP levels have been found in 18 cerebral ischemia patients compared with healthy controls [14]. A new finding of the present work was higher serum levels of SP in non-surviving patients than in surviving patients with severe MMCAI. Moreover, another novel and key finding of our research was the association between serum concentrations of SP and early mortality, after controlling for GCS and age. These findings are in line with another study from our team reporting higher serum concentrations of SP in non-surviving traumatic brain injury (TBI) patients than in surviving patients, and the association between serum concentration of SP and the mortality in those TBI patients [15].

Circulating levels of SP in patients with MMCAI could play a role through different pathways. SP is involved in neurogenic inflammation, which could be stimulated by SP, calcitonin gene-related peptide, and other agents including histamine, serotonin, prostanoids, and leukotrienes. Neurogenic inflammation leads to a painful local inflammatory response that includes the release of neuropeptides, mast cell degranulation, increase in vascular permeability, vasodilation, and the formation of vasogenic edema. Moreover, SP also has a role in the activation of classical inflammation by leukocyte activation, cytokine production, and mast cell activation. Furthermore, SP is involved in the liberation of prostaglandins [16,17], nitric oxide [18], and inflammatory cytokines as interleukin (IL)-1, IL-6, and TNF-alpha [19,20,21,22]. There are data showing that SP is involved in increased vascular permeability and vasogenic edema [23,24,25,26,27]. Besides, the pro-inflammatory cytokines and nitric oxide liberated by SP could activate cellular death by apoptosis. In addition, SP has been associated with vascular thrombosis due to its influence on platelet function, acting as a secondary platelet agonist [28,29]. Thus, all these effects of SP could lead to higher cell loss and finally greater risk of patient death.

Modulators of SP activity in MMCAI patients could have beneficial effects. In some animal models of ischemic stroke, the administration of different NK1 tachykinin receptor antagonists reduced the formation of cerebral edema, permeability of blood-brain barrier (BBB), infarct volume, and functional deficits [30,31,32,33].

Interestingly, opposite findings have been reported by our group with regards to sepsis; surviving severe septic patients showed higher serum concentrations of SP compared to non-survivors [34]. Some animal studies have shown that the SP/NK1R system could be involved in the control of infection, and that its inactivation could lead to poor clearance of bacteria, infectious diseases with more severity, and a lower survival rate [35,36,37].

Our study has some limitations. First, we did not determine other tachykinins. Second, we do not have data on serum concentrations of SP during the follow-up. Third, we did not find a significant association between serum concentrations of SP and TNF-alpha, and we did not explore other possible effects of SP on brain ischemia. Thus, although we were able to conclude that an association between serum concentrations of SP and mortality exists in MMCAI patients, we were unable to conclude that high serum levels of SP are the cause of a poor outcome in MMCAI patients. Fourth, we did not record serum concentrations of SP in healthy subjects; however, the purpose of our study was to compare serum SP levels between non-surviving and surviving patients with MMCAI, and not between healthy subjects and ischemic stroke patients.

## 4. Materials and Methods

### 4.1. Ethical Adherence

Institutional Review Boards of each participating hospital approved this study. Written informed consent from the family members was obtained.

### 4.2. Design and Subjects

This prospective and multi-center study was carried out in 6 Intensive Care Units in Spain. The Institutional Review Board of all participating hospitals approved the study: Hospital General de La Palma (La Palma), Hospital Insular (Las Palmas de Gran Canaria), Hospital Universitario Dr. Negrín (Las Palmas de Gran Canaria), Hospital Universitario Nuestra Señora de Candelaria (Santa Cruz de Tenerife), Hospital Clínico Universitario de Valencia (Valencia), and Hospital Universitario de Canarias (La Laguna, Santa Cruz de Tenerife, Spain). We obtained written informed consent from the legal guardians of all patients.

Inclusion criteria were patients with a severe malignant middle cerebral artery infarction (MMCAI). The diagnosis of severe MMCAI was established with a Glasgow Coma Scale (GCS) [38] lower than 9. Exclusion criteria were: pregnancy, inflammatory or malignant disease, and an age less than 18 years.

The same cohort of patients with severe MMCAI was used in previous publications by our team for other objectives [39,40,41,42]. Previously, we found that serum levels of malondialdehyde [39], soluble CD154 [40], tissue inhibitor of metalloproteinases-1 [41], and total antioxidant capacity [42] are associated with poor prognosis. In the current analysis with 61 severe MMCAI patients, we investigated the association of serum levels of SP with 30-day mortality, including the previously published cohorts with additional patients.

### 4.3. Variables Recorded

The following variables were recorded for all patients: temperature, sodium, platelets, pressure of arterial oxygen (PaO_2_), fraction inspired oxygen (FiO_2_), leukocytes, international normalized ratio, lactic acid, hemoglobin, GCS, fibrinogen, glycemia, creatinine, activated partial thromboplastin time, bilirubin, Acute Physiology and Chronic Health Evaluation II (APACHE II) score [43], age, sex, and decompressive craniectomy. The study end-point was the mortality at 30 days.

### 4.4. Blood Sample Collection

We collected 5 mL of venous blood at diagnosis of severe MMCAI, which were placed in serum separator tubes and centrifuged at 1000× *g* for 15 min after being allowed to clot. Then, serum was frozen at −80 °C.

### 4.5. Substance P (SP) Assay

Determinations of serum levels of SP were carried out at the Institute of Tropical Diseases and Public Health of the Canary Islands, University of La Laguna (Tenerife, Spain) in the Genetic Unit. We assayed serum levels of SP by specific Enzyme Linked Immunosorbent Assay (ELISA) using the kit Substance P Assay (R&D Systems, Abingdon, UK). The limit of detection was 25 pg/mL, and coefficients of variation (CV) were 15% and 9% for inter- and intra-assay, respectively.

### 4.6. Tumor Necrosis Factor (TNF)-Alpha Assay

Determinations of Tumor Necrosis Factor (TNF)-alpha serum levels were carried out in the Laboratory Department, University Hospital of the Canary Islands (La Laguna, Santa Cruz de Tenerife, Spain) using a solid-phase, chemiluminescent immunometric assays kit (Immulite^®^, Siemens Healthcare Diagnostics Products, Llanberis, UK). The limit of detection was 1.7 pg/mL, and CV was <6.5% for interassay.

### 4.7. Statistical Methods

Continuous variables are expressed in the form of medians (interquartile ranges), and categorical variables in the form of frequencies (percentages). We used a Wilcoxon–Mann–Whitney test to compare continuous variables between groups, and a chi-squared test to compare categorical variables.

We used multiple binomial logistic regression analysis to determine the independent association between serum levels of SP levels and mortality at 30 days after controlling for age and GCS. We calculated Odds Ratio and its 95% confidence intervals to measure the impact of each variable.

We constructed a receiver operator characteristic curve with survival at 30 days as a classification variable and serum concentrations of SP as the prognostic variable. We used Kaplan–Meier analysis to determine survival at 30 days in patients showing a cut-off serum levels of SP higher and equal to or lower than 362 pg/mL, and used a log-rank test for the comparison of the distributions of the two samples with respect to survival at 30 days. We used positive likelihood ratio (PLR) to select the cut-off point of serum SP level for 30-day mortality prediction to obtain a balance between sensitivity and specificity. PLR was calculated as sensitivity/100-specificity [44]. In our study, with 77% sensitivity and 60% specificity for the cut-off serum levels of SP of 362 pg/mL, the positive likelihood ratio was 1.93. The traditional axes of false positives (one minus specificity) and true positives (sensitivity) obscure the more important measures of negative and positive likelihood ratios of a diagnostic test. We chose to combine receiver operating characteristic (ROC) curves axes using positive likelihood ratio to obtain the optimal cut-off in the diagnosis test. All *p*-values lower than 0.05 were considered statistically significant. We used the Spearman’s rank correlation coefficient to test the association between serum levels of SP and TNF-alpha. We performed statistical analyses by LogXact 4.1, (Cytel Co., Cambridge, MA, USA), SPSS 17.0 (SPSS Inc., Chicago, IL, USA), MedCalc 15.2.1 (MedCalc Software bvba, Ostend, Belgium), and NCSS 2000 (NCSS, Kaysville, UT, USA).

## 5. Conclusions

Thus, the major novel finding of our study was the association between serum levels of SP and mortality in patients with suffering from severe acute ischemic stroke.

## Figures and Tables

**Table 1:** Biochemical and clinical characteristics of MMCAI patients with respect to 30-day survival.

Characteristics	Survivors (*n* = 30)	Non-Survivors (*n* = 31)	*p* Value
TNF-alpha (pg/mL)—median (p. 25–75)	9.5 (9.1–12.1)	13.0 (10.7–14.4)	0.01
Temperature (°C)—median (p. 25–75)	36.4 (35.6–37.0)	37.0 (36.0–37.4)	0.15
Temperature (°C)—median (p. 25–75)	36.4 (35.6–37.0)	37.0 (36.0–37.4)	0.15
Substance P (pg/mL)—median (p. 25–75)	282 (50–620)	586 (397–990)	<0.001
Sodium (mEq/L)—median (p. 25–75)	139 (136–145)	140 (139–144)	0.42
Platelets—median × 10^3^/mm^3^ (p. 25–75)	218 (171–283)	165 (128–209)	0.003
PaO_2_ (mmHg)—median (p. 25–75)	130 (101–194)	114 (86–153)	0.38
PaO_2_/FiO_2_ ratio—median (p. 25–75)	282 (198–369)	242 (181–325)	0.17
Leukocytes—median × 10^3^/mm^3^ (p. 25–75)	12.8 (9.6–17.1)	13.8 (9.3–18.9)	0.67
Lactic acid (mmol/L)—median (p. 25–75)	1.30 (0.90–1.70)	1.45 (1.00–2.63)	0.18
INR—median (p. 25–75)	1.09 (1.01–1.20)	1.20 (1.04–1.31)	0.11
Hemoglobin (g/dL)—median (p. 25–75)	12.2 (11.4–14.8)	13.7 (11.0–15.0)	0.95
Glycemia (g/dL)—median (p. 25–75)	128 (101–170)	135 (100–159)	0.93
GCS score—median (p. 25–75)	7 (6–8)	6 (3–8)	0.04
Gender female—n (%)	13 (43.3)	11 (35.5)	0.61
Fibrinogen (mg/dL)—median (p. 25–75)	443 (355–518)	417 (323–622)	0.86
Decompressive craniectomy—n (%)	8 (26.7)	5 (16.1)	0.36
Creatinine (mg/dL)—median (p. 25–75)	0.80 (0.60–1.13)	1.00 (0.79–1.23)	0.09
Bilirubin (mg/dL)—median (p. 25–75)	0.65 (0.40–0.93)	0.70 (0.35–1.13)	0.94
aPTT (seconds)—median (p. 25–75)	28 (26–30)	27 (26–33)	0.75
APACHE-II score—median (p. 25–75)	20 (16–25)	22 (19–27)	0.20
Age (years)—median (p. 25–75)	58 (47–67)	64 (53–70)	0.07

PaO_2_ = pressure of arterial oxygen; FiO_2_ = pressure of arterial oxygen/fraction of inspired oxygen; GCS = Glasgow Coma Scale; INR = international normalized ratio; APACHE II = Acute Physiology and Chronic Health Evaluation; aPTT = activated partial thromboplastin time.

**Table 2:** Multiple binomial logistic regression for the prediction of mortality at 30 days.

Variable	Odds Ratio	95% Confidence Interval	*p*
Serum levels of substance P > 362 pg/mL	5.33	1.541–18.470	0.008
Glasgow Coma Scale	0.72	0.529–0.990	0.04
Age (years)	1.03	0.986–1.082	0.17
